# Rituximab treatment in non-lupus full-house nephropathy: A case report

**DOI:** 10.1177/2050313X251333979

**Published:** 2025-05-13

**Authors:** Caroline Gee, Dao Le, Matthew D. Nguyen, Anthony Sisk, Ramy Hanna

**Affiliations:** 1 Division of Nephrology, Hypertension and Kidney Transplantation, University of California, Irvine, CA, USA; 2 Division of Pathology and Laboratory Medicine, University of California, Los Angeles, CA, USA

**Keywords:** non-lupus full house nephropathy, rituximab, immunofluorescence, systemic lupus erythematosus

## Abstract

Non-lupus full-house nephropathy describes a pattern of “full-house” immunofluorescence on renal biopsy in the absence of clinical or serologic markers for systemic lupus erythematosus. It is a rare disease with a broad range of etiologies, including idiopathic, secondary, or a prodrome of systemic lupus erythematosus. Current treatment options are lacking, with mixed results in the literature for varied immunosuppressive regimens. Rituximab, a selective anti-CD20 B-cell monoclonal antibody, has shown success in immune-complex mediated glomerular diseases but has not been commonly used or studied in non-lupus full-house nephropathy. Here, we present a case of non-lupus full-house nephropathy that was refractory to first-line immunosuppressants, in which two rounds of rituximab treatment achieved a positive response. This case emphasizes the need for systemic analysis of rituximab use in non-lupus full-house nephropathy, particularly given its mixed etiologies and presentations.

## Introduction

Non-lupus full-house nephropathy (FHN) is a rare disease that describes the presentation of “full-house” immunofluorescence (IF) in the absence of clinical or serological factors required for systemic lupus erythematosus (SLE) diagnosis.^1^ Full-house IF presents with concurrent positive staining for immunoglobulin A (IgA), immunoglobulin G (IgG), immunoglobulin M (IgM), complement component 3 (C3), and complement component 1q (C1q).^2^ This finding is specific to lupus nephritis in SLE, a systemic autoimmune disorder.^2,3^ However, it can also present independently of SLE based on diagnostic criteria set by the European League Against Rheumatism (EULAR) and the American College of Rheumatology (ACR).^4^ Only a minority of non-lupus FHN cases progress to SLE (with single studies reporting as low as 0%–6%), with the majority being idiopathic or associated with other diseases, termed secondary non-lupus FHN.^5,6^

Current treatment options for non-lupus FHN consist of corticosteroids paired with other immunosuppressants such as chlorambucil, cyclosporine, azathioprine, mycophenolate mofetil (MMF), and cyclophosphamide.^7–9^ Experimental studies suggest B-cell activation may lead to immunoglobulin deposition in the glomerular basement membrane, resulting in renal dysfunction and proteinuria.^10^ Therefore, there has been growing interest in the use of B-cell depletion for immune complex-mediated glomerular diseases. B-cell depletion is currently utilized in other autoimmune diseases, including idiopathic membranous nephropathy, SLE, and rheumatoid arthritis, but often with nonselective agents, such as cyclophosphamide, which may pose harmful side effects.^11^

Rituximab is a chimeric monoclonal antibody that selectively targets the CD20 antigen on B cells. Rituximab was approved by the Food and Drug Administration in 1997 for non-Hodgkin’s lymphoma and has since been used in an array of autoimmune diseases, including SLE with lupus nephritis and rheumatoid arthritis.^12,13^ The benefits of rituximab include its selective B-cell depletion, as the CD20 antigen is not found on other plasma cells or normal tissue. Rituximab has been increasingly used in primary glomerular diseases, including idiopathic membranous nephropathy, minimal change disease, and focal segmental glomerulosclerosis, with mixed success.^11,14–16^

Here, we present a patient with non-lupus FHN who was refractory to first-line immunosuppressants, in which treatment with rituximab achieved a positive response.

## Case presentation

A 58-year-old Hispanic female with a history of hypertension (HTN) was referred to our clinic for evaluation of proteinuria and hematuria. She had no history of renal conditions. The patient’s medications included amlodipine 10 mg daily for HTN as well as nonsteroidal anti-inflammatory drugs and colchicine for ongoing “gout flares,” with uric acid 9.8 mg/dL (however, she did not have any formal gout diagnosis by needle centesis). Incidentally, she had contracted COVID-19 the year prior. The patient had a body mass index of 31.46 kg/m² (height 5′3″, weight 80.6 kg), and vitals were within normal limits. Physical exam was benign without lower extremity edema.

Initial laboratory workup showed 24-h urine protein 7.2 g/24 h, 24-h urine creatinine 50.4 mg/dL, and urine protein-to-creatinine ratio (UPCR) 5.3 g/g. She had serum creatinine 1.7 mg/dL, blood urea nitrogen (BUN) 42 mg/dL, and an estimated glomerular filtration rate (eGFR) of 31–38 mL/min/1.73 m^2^. Urinalysis showed proteinuria >500 mg/dL, small hemoglobin, and 12 RBCs million/µL. Serologies were negative for SLE or other autoimmune conditions, including a negative work-up for antinuclear antibodies (ANA), antineutrophil cytoplasmic antibodies, C3/C4, anti-double-stranded DNA, anti-Smith, phospholipase A2 receptor, thrombospondin type-1 domain-containing 7A, and cryoglobulins. Electrophoresis and immunofixation studies were negative for monoclonal gammopathy. The patient was found to be positive for hepatitis B core IgG but had no active infection, with a negative viral load by polymerase chain reaction and negative hepatitis B surface IgG antigen. She was negative for hepatitis A/C and human immunodeficiency virus (HIV). Renal ultrasound was normal, with no sonographic evidence of chronic kidney disease or hydronephrosis. Baseline complete blood count showed platelets 259 thousand/µL and normocytic anemia of chronic disease at baseline: hemoglobin 9.8 g/dL with a mean corpuscular volume of 91.0 fL. The patient also had low white blood cells (WBCs) at 3.8 thousand/µL, but this improved to 4.3 thousand/µL on repeat check 3 weeks later.

**Figure 1.**
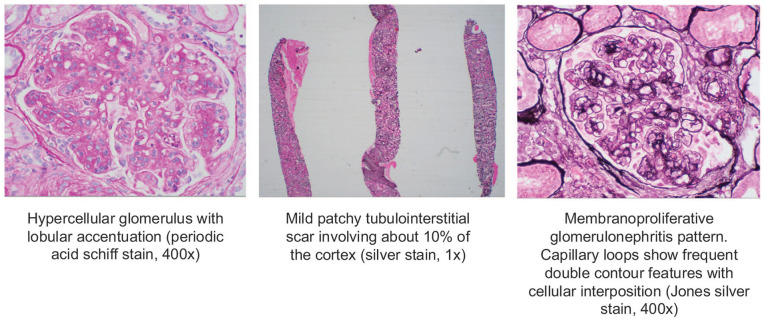
Renal biopsy histology images. No necrosis or crescent formation seen. No segmental sclerosis.

**Figure 2.**
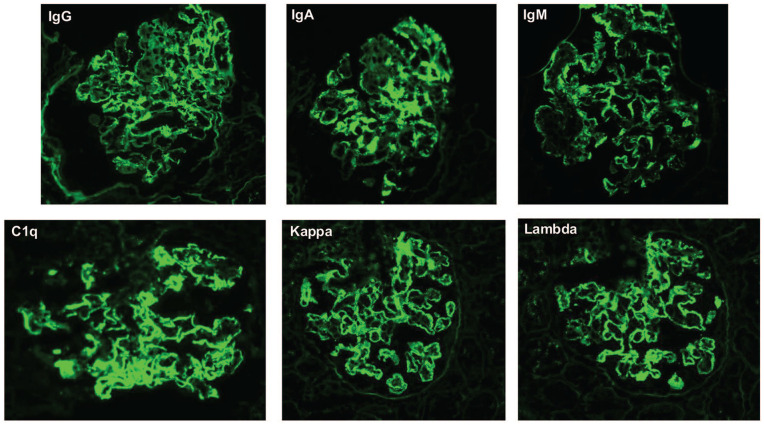
Renal biopsy immunofluorescence reveals a “full-house” pattern with global mesangial and capillary loop staining for IgG (2–3+), IgA (3–4+), IgM (3–4+), C3 (2+; not pictured), C1q (3–4+), kappa (3+), and lambda (3+; all 400×). C1q: complement component 1q; C3: complement component 3; IgA: immunoglobulin A; IgG: immunoglobulin G; IgM: immunoglobulin M.

**Figure 3.**
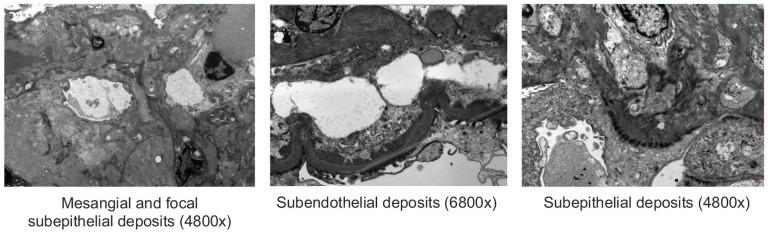
Renal biopsy transmission electron microscopy images of corticomedullary tissue. No tubuloreticular inclusions are seen.

Renal biopsy was obtained, which demonstrated immune complex-associated membranoproliferative glomerulonephritis (MPGN; Figure 1). IF showed full-house staining: IgG (2–3+), IgA (3–4+), IgM (3–4+), C3 (2+), C1q (3–4+), kappa (3+), and lambda (3+) (Figure 2). There was a mild tubulointerstitial scar (10%) and mild arterial sclerosis. No segmental sclerosis. Electron microscopy showed subepithelial and intramembranous deposits and large subendothelial deposits. No tubuloreticular inclusions (TRIs) were seen (Figure 3).

The patient was started on prednisone 40 mg daily for 1 month, with plans to taper down over 2–3 months, along with atovaquone 1500 mg daily for pneumocystis jirovecii pneumonia (PJP) prophylaxis. She was seen by rheumatology, who agreed that the patient’s presentation did not include any signs of SLE or systemic autoimmune etiology. The patient was also seen by infectious disease for re-vaccination of hepatitis B, with confirmed immunity on antibody panels thereafter.

At her 3-month follow-up, the patient’s renal function had not improved on prednisone 10 mg alone, with UPCR 10.4 g/g. Labs showed serum creatinine 1.9 mg/dL, eGFR 27–33 mL/min/1.73 m^2^, and BUN 44 mg/dL. Given the lack of improvement, azathioprine was added, starting at 25 mg daily with a 1-month increase to 50/75 mg on alternating days. The patient’s anemia had worsened, with hemoglobin 8.9 g/dL (down from 9.5 g/dL 1 month prior). The patient was then started on monthly darbepoetin alfa 25 µg injections and daily oral ferrous sulfate 142 mg with a hemoglobin goal of 10–11 g/dL.

At the 5-month follow-up, the patient only had minimal improvements in renal function with prednisone and azathioprine, with UPCR 9.1 g/g, creatinine 2.1 mg/dL, and eGFR 24–29 mL/min/1.73 m^2^. At this time, the decision was made to switch to rituximab. The patient was started on tenofovir for hepatitis B reactivation prophylaxis and atovaquone for PJP prophylaxis. Due to continually downtrending creatinine clearance, with a low of 29 mL/min, she was then switched from tenofovir to entecavir 0.5 mg every 72 h (so long as creatinine clearance remained between 10 and 30 mL/min).

Three months later, the patient received her first round of intravenous rituximab 1000 mg, with two doses spaced 2 weeks apart. At her 3-month follow-up, the patient demonstrated improved serum creatinine of 2.1 mg/dL (down from 2.8 mg/dL 3 months prior), along with BUN 49 mg/dL and eGFR 24–29 mL/min/1.73 m^2^ (compared to 3 months prior studies showing BUN 54 mg/dL and eGFR 17–21 mL/min/1.73 m^2^). Twenty-four hours urine protein was 10.3 g/24 h, down from 11.8 g/24 h 3 months prior, and UPCR improved to 8.35 g/g, down from 10.4 g/g 3 months prior. Her proteinuria further improved to 24-h urine protein 5.46 g/24 h and UPCR 5.77 g/g 1 month later. The patient’s WBC count was notably low at 3.4 thousand/µL, down from 4.9 thousand/µL when she received rituximab 3 months prior, but the patient confirmed she had been off azathioprine for 6 months by then. Hemoglobin had only improved to 9.4 g/dL, so darbepoetin alfa was increased to 40 µg monthly.

**Figure 4.**
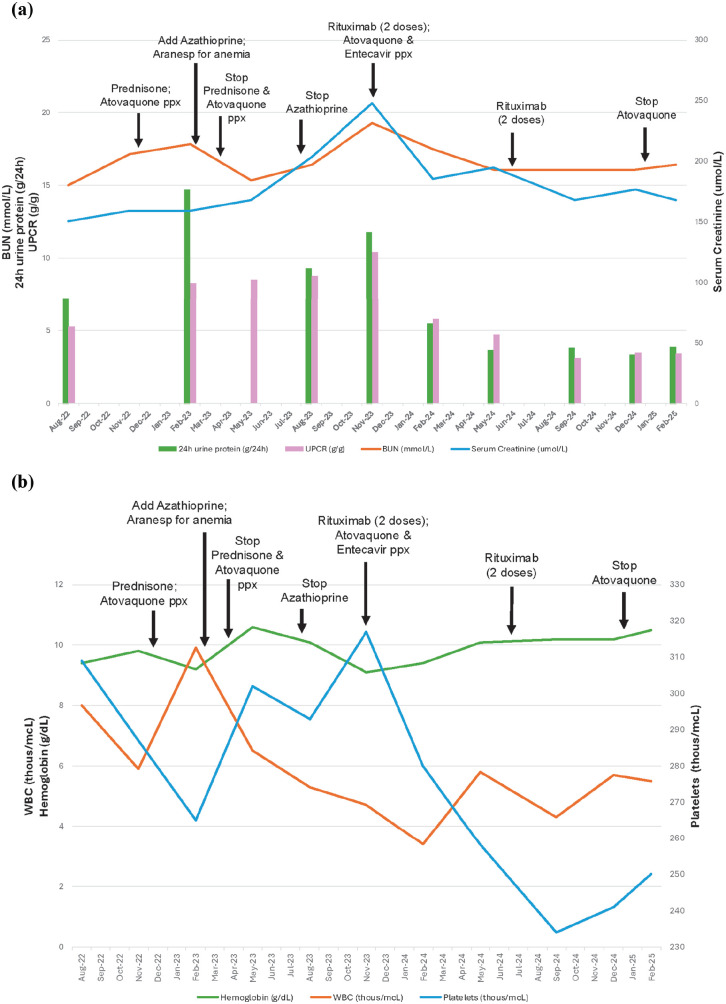
Progression with intervention timestamps. (a) Renal function. (b) Hematologic stability. BUN: blood urea nitrogen; Ppx: prophylaxis; UPCR: urine protein-to-creatinine ratio; WBC: white blood cell.

The patient received her second round of rituximab 1000 mg (two infusions spaced 2 weeks apart) 6 months later. At her 2-month follow-up, the patient demonstrated a significant response with 24-h urine protein 3.8 g/24 h and UPCR 3.1 g/g, as seen in Figure 4(a). Basic metabolic panel showed renal improvement with serum creatinine 1.9 mg/dL, BUN 45 mg/dL, and eGFR 27–33 mL/min/1.73 m^2^. WBCs were stable at 4.3 thousand/µL, with no recent infections. Hemoglobin improved to 10.2 g/dL on darbepoetin alfa, with stable RBCs 3.31 million/µL, hematocrit 30.3%, and platelets 234 thousand/µL, as seen in Figure 4(b). Repeat serologies remained negative for SLE, including negative ANA, ds-DNA, and C3/C4 complements, and repeat electrophoresis remained negative for paraproteinemia. At follow-up 3 months later, the patient showed serum creatinine 2.0 mg/dL, BUN 45 mg/dL, and eGFR 28 mL/min/1.73 m^2^. Urine studies showed UPCR of 3.47 g/g and 24-h urine protein of 3.3 g/24 h. Her creatinine clearance was 36 mL/min, so entecavir was decreased to every 48 h (given creatinine clearance remained between 30 and 50 mL/min). Atovaquone was also discontinued as the patient no longer had leukopenia with WBCs 5.7 thousand/µL.

At follow-up 2 months later, the patient demonstrated continued positive response with UPCR 3.4 g/g and 24-h urine protein 3.9 g/24 h. Her serum creatinine was 1.9 mg/dL and BUN 46 mg/dL, but her eGFR improved to 30 mL/min/1.73 m^2^. Since the initiation of rituximab 14 months prior, the patient achieved a >50% reduction in proteinuria, from UPCR 10.4 g/g and 24-h urine protein 11.8 g/24 h to 3.4 g/g and 3.9 g/24 h, respectively (Table 1). The decision was made to hold rituximab unless UPCR exceeds 3.5 g/g, in the context of a lack of formal guidelines for this rare disease. The patient will be carefully monitored to determine if additional rituximab is indicated.

**Table 1.** Summary of laboratory values.

	Aug 2022	Nov 2022	Feb 2023	May 2023	Aug 2023	Nov 2023	Feb 2024	May 2024	Sept 2024	Dec 2024	Feb 2025
SCr (mg/dL)	1.7	1.8	1.8	1.9	2.3	2.8	2.1	2.2	1.9	2	1.9
BUN (mg/dL)	42	48	50	43	46	54	49	45	45	45	46
eGFR (mL/min/1.73 m^2^)	34.5	32	32	30	24.5	19	26.5	25.5	30	28	30
24 h urine protein (g/24 h)	7.24		14.7		9.33	11.8	5.46	3.65	3.81	3.31	3.86
UPCR (g/g)	5.27		8.24	8.52	8.77	10.4	5.77	4.73	3.1	3.47	3.4
Hemoglobin (g/dL)	9.4	9.8	9.2	10.6	10.1	9.1	9.4	10.1	10.2	10.2	10.5
Platelets (thousands/µL)	309	287	265	302	293	317	280	258	234	241	250
WBC (thousands/µL)	8	5.9	9.9	6.5	5.3	4.7	3.4	5.8	4.3	5.7	5.5

BUN: blood urea nitrogen; eGFR: estimated glomerular filtration rate; SCr: serum creatinine; UPCR: urine protein-to-creatinine ratio; WBC: white blood cell.

## Discussion

In this case study, we presented a case of non-lupus FHN, in which rituximab achieved sustained reductions in proteinuria. Our patient did not meet the EULAR/ACR criteria for SLE, which specifies an obligatory positive ANA along with meeting weighted criteria in seven clinical and three immunologic (antiphospholipid antibodies, complement proteins, and SLE-specific antibodies) categories.^4^

Non-lupus FHN has been classified as idiopathic, prodromal to SLE, or secondary (due to membranous nephropathy, IgA nephropathy, infection-related glomerulonephritis, or anti-neutrophil cytoplasmic antibody-associated glomerulonephritis).^5^ The spectrum of non-lupus FHN presentation was first described by Wen and Chen among 24 patients. The cohort is classified into six categories: membranous glomerulonephritis (46%), IgA nephropathy (21%), MPGN (12.5%), post-infectious glomerulonephritis (12.5%), C1q nephropathy (4%), and unclassified mesangial (4%). None of the patients in their study had TRIs. Only two patients (8.3%) developed serological and clinical signs of SLE within a 14-month follow-up.^1^ Other studies have demonstrated the progression of idiopathic FHN to SLE over a longer follow-up period of up to 14 years. Still, in the literature, only a minority of patients with non-lupus FHN go on to develop clinical or serological signs of SLE.^5,6,17,18^

The patient in our case presented with idiopathic non-lupus FHN and did not meet any clinical or serological criteria for SLE diagnosis, including over a more than 2-year follow-up period. Serologies were negative for any other secondary disease process, such as primary membranous nephropathy or viral etiology. Our patient displayed membranoproliferative findings that were negative for C3 glomerulonephritis (C3GN) or dense deposit disease (DDD). It is possible that our patient’s renal findings represent lupus nephritis as a prodrome of SLE, but this would likely require several years of follow-up to elucidate. Of note, FHN with TRIs has been more highly associated with lupus nephritis and subsequent SLE development, but this was not present on our patient’s biopsy.^18^

Other reported pathologies associated with FHN include liver disease, diabetes mellitus, IgA nephropathy, C1q nephropathy, and infections, including HIV and hepatitis B or C.^19,20^ Our patient did not have any active signs of the above diseases. While our patient was found to have a positive hepatitis B core IgG antigen without active infection, she had confirmed immunity on repeat antibody labs. Chronic hepatitis B and C infections have been associated with MPGN, but it is unlikely to be the etiology in this patient’s case, given she did not have an active infection.^21^ Of note, this patient did have a COVID-19 infection 1 year prior to her presentation. Sethi et al. described a case of non-lupus FHN following COVID-19 infection; however, the patient also had preexisting focal segmental glomerulosclerosis secondary to a SMARCAL1 gene mutation, with unknown contributions. In addition, most COVID-19-associated renal biopsies describe acute tubular necrosis and glomerular lesions from collapsing glomerulopathy.^22–24^ While this patient’s prior COVID-19 infection could be related, we do not suspect it is a driving factor in her renal etiology.

To our knowledge, there is currently no standardized, evidence-based treatment approach for non-lupus FHN. Treatment options typically mimic those for lupus nephritis, consisting of steroids with immunosuppressants such as cyclophosphamide, azathioprine, cyclosporine A, or MMF. Success rates vary in the literature, with some patients achieving significant partial or complete remission while others progress to end-stage renal disease.^7–9,19,25,26^ The type of glomerular disease in FHN may also require different treatments. de Oliveira Silva et al. described a case of three patients with non-lupus FHN who were treated with corticosteroid therapy with cyclophosphamide. Two patients with proliferative glomerulonephritis progressed to renal failure, while the third patient with membranous nephropathy achieved remission.^19^

Rituximab is a chimeric monoclonal antibody that selectively binds the CD20 receptor of B cells.^12^ It has been demonstrated to achieve partial or complete sustained remission of idiopathic membranous nephropathy.^14,27^ It has also been used with some success in lupus nephritis and immunoglobulin-associated MPGN.^13,16,28^ However, there are limited studies on the use of rituximab for non-lupus FHN specifically. Sadineni and Das described a case of a patient with non-lupus FHN who achieved partial remission with rituximab. The patient, who presented with membranous nephropathy on biopsy, failed to respond to oral steroids, MMF, or tacrolimus. Unfortunately, the dosing regimen used for rituximab was not stated, and all other patients in the study were treated with more common immunosuppressants.^9^ Prudhvi et al. described achieving partial remission with rituximab in “lupus-like membranous nephropathy” in a patient with well-controlled HIV infection who did not meet SLE criteria. The patient in this case presented with immune-complex glomerulonephritis, notably with numerous subepithelial and paramesangial deposits and TRIs on electron microscopy (EM). After not responding to lisinopril alone, the patient received rituximab 1000 mg intravenously spaced 2 weeks apart, after which she achieved partial remission over 4 months.^29^ Given the lack of literature on rituximab’s use in non-lupus FHN, there is no established indication of when rituximab would be specifically recommended over other agents or contraindicated for this disease. However, rituximab is generally contraindicated in cases where immunosuppressive therapy is inappropriate, such as active hepatitis B infection, active tuberculosis, septic shock, or bacteremia.^30^

Our patient was not responsive to prednisone alone or in conjunction with azathioprine, which are common treatment options for non-lupus FHN. However, she demonstrated a positive response to rituximab over the course of 14 months, with a >50% reduction in proteinuria that was sustained 6 months beyond her last treatment. With limited data on rituximab’s use in non-lupus FHN, the dosing regimen for rituximab was primarily based on prior studies that used the same regimen with success in idiopathic membranous nephropathy and MPGN.^11,16,29^ The decision was made to hold rituximab unless the patient’s UPCR increases above 3.5 g/g into the nephrotic range (currently UPCR 3.4 g/g). She will continue to be closely monitored to assess the need for additional rounds of rituximab.

Through selective B-cell depletion, rituximab likely reduced immune complex-mediated complement activation and resultant glomerular and tubular damage in our patient. Rituximab has also been shown to have a protective effect on podocytes through stabilizing the actin cytoskeleton to prevent apoptosis. Given that FHN follows variable presentations, it is unclear if rituximab will have similar efficacies in all subtypes, but it targets the shared feature of immune complex deposition.^11,16^

This case suggests that rituximab may be considered as a treatment option for non-lupus FHN in cases that are refractory to first-line immunosuppressants. Given its selective action, rituximab may be considered earlier in the treatment plan to avoid the more aggressive side effect profiles of other immunosuppressants. Further systemic studies are needed to elucidate how rituximab may function in different types of non-lupus FHN given its broad range of etiologies.

## Conclusion

Non-lupus FHN is a rare, understudied disease with a range of possible etiologies that may manifest only years after initial renal symptoms develop. There are no standardized treatment regimens, with mixed success rates for different permutations of immunosuppressive regimens. Our patient was refractory to first-line immunosuppressant medications but demonstrated a positive response to two rounds of rituximab given over 14 months. Future studies are needed to elucidate the long-term effects of rituximab in non-lupus FHN and its ability to address a broad range of clinical presentations.
